# Preconception depression reduces fertility: a couple-based prospective preconception cohort

**DOI:** 10.1093/hropen/hoae032

**Published:** 2024-05-16

**Authors:** Tierong Liao, Yaya Gao, Xinliu Yang, Yanlan Tang, Baolin Wang, Qianhui Yang, Xin Gao, Ying Tang, Kunjing He, Jing Shen, Shuangshuang Bao, Guixia Pan, Peng Zhu, Fangbiao Tao, Shanshan Shao

**Affiliations:** Department of Maternal, Child and Adolescent Health, School of Public Health, Anhui Medical University, Hefei, China; Department of Maternal, Child and Adolescent Health, School of Public Health, Anhui Medical University, Hefei, China; Anhui Provincial Key Laboratory of Population Health and Aristogenics, Anhui Medical University, Hefei, China; Anhui Provincial Key Laboratory of Population Health and Aristogenics, Anhui Medical University, Hefei, China; Key Laboratory of Population Health Across Life Cycle, Ministry of Education of the People’s Republic of China, Anhui Medical University, Hefei, China; Key Laboratory of Population Health Across Life Cycle, Ministry of Education of the People’s Republic of China, Anhui Medical University, Hefei, China; Key Laboratory of Population Health Across Life Cycle, Ministry of Education of the People’s Republic of China, Anhui Medical University, Hefei, China; Key Laboratory of Population Health Across Life Cycle, Ministry of Education of the People’s Republic of China, Anhui Medical University, Hefei, China; Department of Maternal, Child and Adolescent Health, School of Public Health, Anhui Medical University, Hefei, China; Department of Maternal, Child and Adolescent Health, School of Public Health, Anhui Medical University, Hefei, China; Key Laboratory of Population Health Across Life Cycle, Ministry of Education of the People’s Republic of China, Anhui Medical University, Hefei, China; Department of Epidemiology and Biostatistics, School of Public Health, Anhui Medical University, Hefei, China; Department of Maternal, Child and Adolescent Health, School of Public Health, Anhui Medical University, Hefei, China; Key Laboratory of Population Health Across Life Cycle, Ministry of Education of the People’s Republic of China, Anhui Medical University, Hefei, China; Department of Maternal, Child and Adolescent Health, School of Public Health, Anhui Medical University, Hefei, China

**Keywords:** depression, infertility, time to pregnancy, couple, conception, cohort

## Abstract

**STUDY QUESTION:**

Is preconception depression associated with time to pregnancy (TTP) and infertility?

**SUMMARY ANSWER:**

Couples with preconception depression needed a longer time to become pregnant and exhibited an increased risk of infertility.

**WHAT IS KNOWN ALREADY:**

Preconception depression in women contributes to impaired fertility in clinical populations. However, evidence from the general population—especially based on couples—is relatively scant.

**STUDY DESIGN, SIZE, DURATION:**

A couple-based prospective preconception cohort study was performed in 16 premarital examination centers between April 2019 and June 2021. The final analysis included 16 521 couples who tried to conceive for ≤6 months at enrollment. Patients with infertility were defined as those with a TTP ≥12 months and those who conceived through ART.

**PARTICIPANTS/MATERIALS, SETTING, METHODS:**

Couples’ depression was assessed using the Patient Health Questionnaire-9 at baseline. Reproductive outcomes were obtained via telephone at 6 and 12 months after enrollment. Fertility odds ratios (FORs) and infertility risk ratios (RRs) in different preconception depression groups were analyzed using the Cox proportional-hazard models and logistic regression, respectively.

**MAIN RESULTS AND THE ROLE OF CHANCE:**

Of the 16 521 couples analyzed, 10 834 (65.6%) and 746 (4.5%) couples achieved pregnancy within the first 6 months and between the 6th and 12th months, respectively. The median (P_25_, P_75_) TTP was 3.0 (2.0, 6.0) months. The infertility rate was 13.01%. After adjusting for potential confounders, in the individual-specific analyses, we found that preconception depression in women was significantly related to reduced odds of fertility (FOR = 0.947, 95% CI: 0.908–0.988), and preconception depression in either men or women was associated with an increased risk of infertility (women: RR = 1.212, 95% CI: 1.076–1.366; men: RR = 1.214, 95% CI: 1.068–1.381); in the couple-based analyses, we found that—compared to couples where neither partner had depression—the couples where both partners had depression exhibited reduced fertility (adjusted FOR = 0.904, 95% CI: 0.838–0.975). The risk of infertility in the group where only the woman had depression and both partners had depression increased by 17.8% (RR = 1.178, 95% CI: 1.026–1.353) and 46.9% (RR = 1.469, 95% CI: 1.203–1.793), respectively.

**LIMITATIONS, REASONS FOR CAUTION:**

Reporting and recall bias were unavoidable in this large epidemiological study. Some residual confounding factors—such as the use of anti-depressants and other medications, sexual habits, and prior depressive and anxiety symptoms—remain unaddressed. We used a cut-off score of 5 to define depression, which is lower than prior studies. Finally, we assessed depression only at baseline, therefore we could not detect effects of temporal changes in depression on fertility.

**WIDER IMPLICATIONS OF THE FINDINGS:**

This couple-based study indicated that preconception depression in individuals and couples negatively impacts couples’ fertility. Early detection and intervention of depression to improve fertility should focus on both sexes.

**STUDY FUNDING/COMPETING INTEREST(S):**

This work was supported by grants from the National Natural Science Foundation of China (No. 82273638) and the National Key Research and Development Program of China (No. 2018YFC1004201). All authors declare no conflicts of interest.

**TRIAL REGISTRATION NUMBER:**

N/A.

WHAT DOES THIS MEAN FOR PATIENTS? Studies have demonstrated that depressive symptoms in either women or men are associated with decreased fertility in the clinical population. However, these conclusions are challenging to extrapolate to the general population. Additionally, few studies have explored the combined effects of depressive symptoms in men and women on couples’ fertility. Therefore, this study used a community-based cohort of 16 521 couples trying to conceive to explore the individual and combined effects of depressive symptoms in men and women on time to pregnancy (TTP) and infertility. The results revealed that, irrespective of the other partner’s depressive symptoms, preconception depression in either men or women was associated with an increased risk of infertility, and preconception depression in women was associated with a longer TTP. When considering the other partner’s depressive symptoms, compared to couples where neither partner had depression, couples where both partners had depression needed a longer time to become pregnant, and couples where both partners or only the woman had depression exhibited an increased risk of infertility; however, the associations between depression and TTP and infertility were not observed in couples where only the man had depression. For patients and policymakers, these results support that screening for depression in couples who are actively preparing for pregnancy and providing psychological intervention for those with positive screening results are important. These measures will help reduce the TTP and incidence of infertility, especially for couples where both men and women suffer from depression and women are under 30 years old.

## Introduction

Infertility—defined as the failure to establish a clinical pregnancy after 12 months of regular and unprotected sexual intercourse—is estimated to affect 8–12% of reproductive-aged couples worldwide (Vander Borght and Wyns, 2018). Infertility harms the physical and mental health of couples attempting to conceive (Shani *et al.*, 2016; Salih Joelsson *et al.*, 2017; Dokras *et al.*, 2018) and also contributes to a massive worldwide illness burden (Sun *et al.*, 2019). Despite ART having become more available, effective, and safe in recent years (Bai *et al.*, 2020), the overall clinical pregnancy rate is still low (Chambers *et al.*, 2021). Thus, identifying the risk factors for infertility is of great significance for public health. Recently, some lifestyle factors—such as physical activity, eating habits, and sleep duration—and environmental factors have been found to affect reproductive capacity (Carré *et al.*, 2017; Massányi *et al.*, 2020; Mena *et al.*, 2020; Skoracka *et al.*, 2021; Hernáez *et al.*, 2022; Yao *et al.*, 2022). Additionally, the influence of psychological factors (e.g. depression), which are often intertwined with lifestyle factors, on infertility has been debated for years (Rooney and Domar, 2018; Difrancesco *et al.*, 2019; Vancampfort *et al.*, 2021; Korczak *et al.*, 2022), but the results remain largely inconclusive (Williams *et al.*, 2007).

Depression is a widespread chronic illness characterized by low mood, lack of energy, sadness, insomnia, and anhedonia (Cui, 2015). Estimates suggest that 14% of women suffer from depression during their reproductive years and that women are disproportionately more prone to it than men (Weissman and Olfson, 1995; Smith, 2014). Depressive symptoms may reduce fertility in several ways, such as by disrupting the hypothalamic–pituitary–gonadal (HPG) axis, which affects sex hormone production; and by reducing libido, sexual intercourse frequency, and sleep quality (Sansone *et al.*, 2018; Meller *et al.*, 2001; Hernáez *et al.*, 2022; Yao *et al.*, 2022; Zhu *et al.*, 2022). Thus far, numerous studies have explored the relationship between depressive symptoms and fertility. Some cross-sectional studies have suggested that depressive symptoms are more common among couples who are infertile than those who are fertile (Pasch *et al.*, 2016); one study reported that 39.1% of women and 15.3% of men fulfilled the criteria for major depressive disorder during infertility treatment (Holley *et al.*, 2015). According to meta-analytic data from prospective cohort studies, depression and state anxiety scores during ART are associated with poor outcomes (Purewal *et al.*, 2018). In randomized clinical trials of patients with infertility, those who received psychological interventions exhibited greater odds of becoming pregnant than those who did not (control group) (Hämmerli *et al.*, 2009), suggesting that an improvement in mental health is beneficial for enhancing fertility. However, most of these studies were based on couples seeking assisted reproductive assistance, who have already endured a long period of struggling with pregnancy difficulties at the time of their evaluation of depressive symptoms (Williams *et al.*, 2007). Therefore, whether infertility contributes to depression or depression contributes to infertility is unclear.

A growing body of studies has examined the effect of preconception depression on fertility in the non-ART population. However, most of them have measured depression solely in women (Lynch *et al.*, 2012; Nillni *et al.*, 2016), and few studies have considered depression in men in the context of assessing couples’ fertility. Prior research has indicated that depressive symptoms in men are associated with poor semen parameters, including low semen volume, low total sperm count, and low total motility (Yland *et al.*, 2021; Ye *et al.*, 2022), which probably translate into low couple fertility. Thus far, several studies have explored the relationship between depression and fertility in men and women, as well as in couples. However, these analyses solely focused on individuals, not couples, with their results demonstrating considerable heterogeneity. For example, a register-based study of 1 408 951 individuals in Finland found that depression is associated with a lower likelihood of having children and having fewer children, among both men and women (Golovina *et al.*, 2023). However, another national-registry-based study found no link between depression in women and the number of children that they had (Power *et al.*, 2013). In contrast, a prospective cohort study conducted among couples where the woman had been diagnosed with PCOS or unexplained infertility reported that depression is related to slightly decreased odds of pregnancy among men but not women (Evans-Hoeker *et al.*, 2018).

To our knowledge, no study has explored the association between couples’ depression and their fertility. However, couples, as a whole, usually share a common living environment, some lifestyle and health behaviors, and emotions. Research has indicated a significant interdependence between health behaviors and depressive symptoms among partners in a couple (Yang *et al.*, 2023). Depressive symptoms in one partner significantly impact depressive symptoms in the other (Tower and Kasl, 1995), which is also supported by data from our previous study (Gan *et al.*, 2022). However, the ability to conceive depends on the couple, not merely the man or the woman. Thus, it is worthwhile to assess the joint effects of depressive symptoms in both partners to arrive at an improved understanding of depression’s effect on fertility among couples.

In this couple-based prospective cohort study, we examined the effects of couples’ preconception depression on their fertility (time to pregnancy [TTP] and infertility). We aimed to explore: the associations between each partner’s depression and couple fertility; effects of co-exposure to depression among couples on couple fertility; and potential moderating factors of the association between preconception depression and couple fertility. We assessed the participants’ depression during the period of preparing for pregnancy and, subsequently, followed up on their reproductive outcomes, to generate evidence for causal inference of depression on fertility.

## Materials and methods

### Study design and population

**Figure 1.**
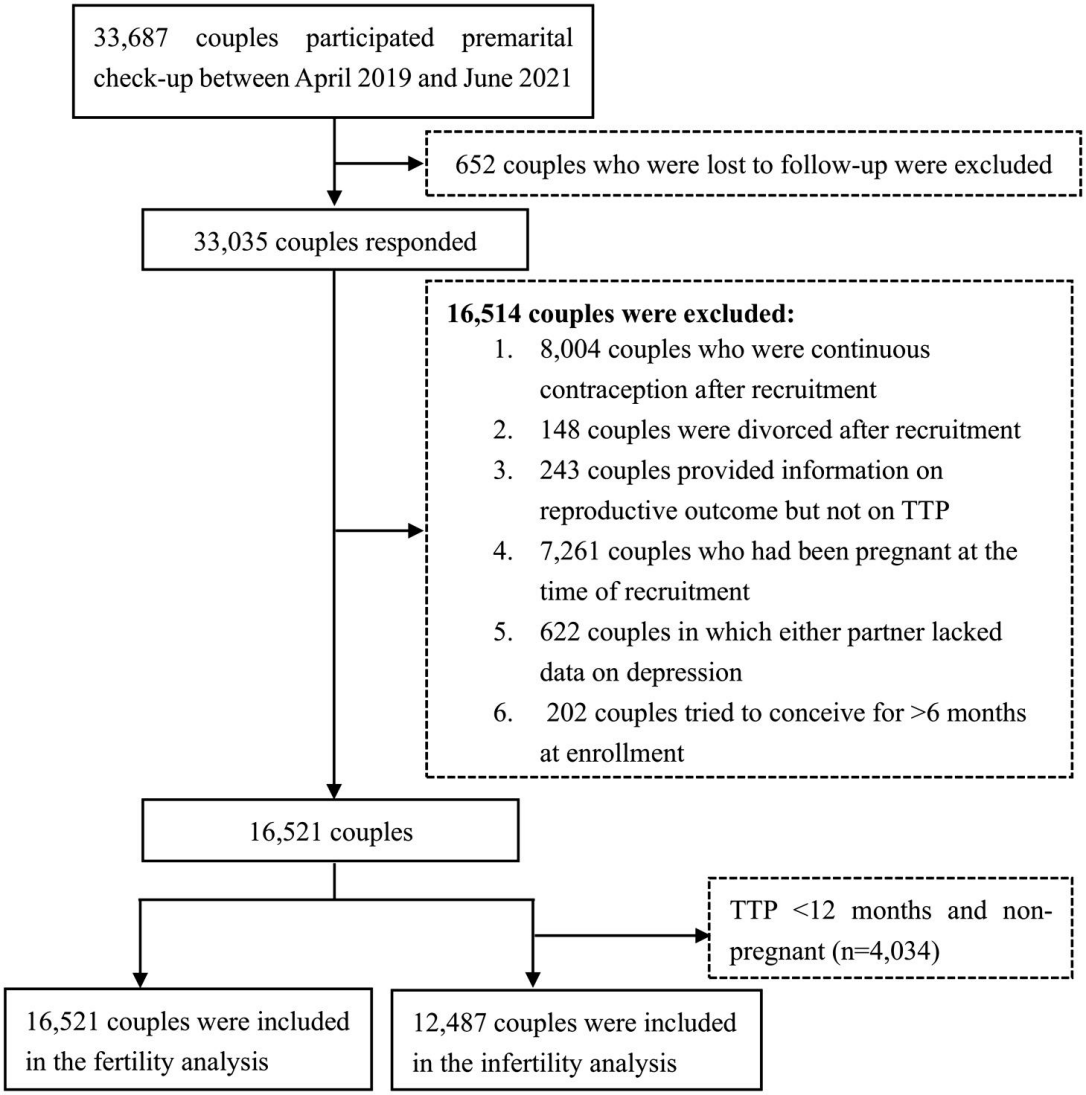
**Flow chart for the study population to examine the effects of couples’ preconception depression on their fertility.** TTP, time to pregnancy.

We conducted a couple-based reproduction cohort study by employing the Reproductive Health of Childbearing Couple-Anhui Cohort (RHCC-AC), which aimed to investigate the effect of psychological and environmental factors on fertility among couples of childbearing age. Overall, 33 687 couples were recruited as participants at 16 premarital examination centers in 16 cities/counties in China’s Anhui Province from April 2019 to June 2021. At enrollment, all participants completed a baseline questionnaire including information regarding their demographic characteristics, lifestyle, psychological condition, and diet, and provided their biological samples (e.g. blood, urine). Each couple was followed up by telephone at 6 and 12 months after enrollment to obtain their fertility status. In our study, patients with infertility are defined as those with a TTP ≥12 months and those who conceived through ART. Couples who could not be contacted or refused to respond at each follow-up visit were considered lost to follow-up and were excluded from this study (n = 652). Additionally, 16 514 couples were excluded for other reasons (Fig. 1). Finally, we included 16 521 and 12 487 couples in the fertility and infertility analyses, respectively. Overall, 4034 couples with TTP <12 months and not pregnant were excluded from the infertility analysis.

The study was approved by the Ethics Committees of Anhui Medical University (approval number: 20189999) and was conducted in accordance with the Helsinki Declaration. All the participants provided informed consent.

### Measures

#### Depressive symptoms (exposure)

At enrollment, participants filled the Patient Health Questionnaire-9 (PHQ-9), which has been recommended as a screening measure for depression in primary care (Zimmerman, 2019). It includes nine items that assess the frequency of the corresponding symptoms that they have experienced over the past 2 weeks. Each item is rated on a four-point ordinal scale—specifically, ‘0 = seldom or never’, ‘1 = a few days’, ‘2 = more than half of the days’, and ‘3 = nearly every day’. The total score (ranging from 0–27) of depressive symptoms is computed by summing the ratings on the nine symptom items, with higher scores indicating a greater degree of depressive symptoms. In this study, we defined a PHQ-9 cut-off score of ≥5 as depression, which exhibited a specificity and sensitivity of 0.75 and 0.86, respectively, in primary care (Zuithoff *et al.*, 2010). For the individual-specific analyses, we divided the partners into the following two groups: no-depression; and depression. For the couple-based analyses, we divided the couples into the following four groups: neither partner with depression; man-only depression; woman-only depression; and both partners with depression. The Cronbach’s α-coefficient of the nine items for women and men were 0.807 and 0.800, respectively.

#### Fertility (outcome)

We used TTP (continuous variable) and infertility (dichotomous variable) as indicators to assess the couples’ fertility. All couples were contacted by the medical staff or researcher at 6 and 12 months after enrollment to obtain information regarding their pregnancy outcome, contraceptive status, and abortion as well as the time of the outcome’s occurrence. The pregnancy outcome was ascertained using a single-choice question, specifically ‘From recruitment to now, have you been pregnant?’ The response alternatives were as follows: ‘1 = Yes’, ‘2 = No’, and ‘3 = Yes but miscarried’. In this study, pregnancy was defined as a clinical pregnancy, which must be confirmed by a pelvic ultrasound scan. For the couples who responded ‘Yes’ or ‘Yes but miscarried’, we further asked the following questions to obtain the TTP: ‘How long (in months) did it take you to become pregnant having regular sex without using any contraception?’ For those who responded ‘No’, we asked the following questions to obtain the TTP: ‘How long have you and your partner been having regular sex without contraception (in months) in order to become pregnant?’ Importantly, the duration of pregnancy attempts before recruitment was also included in calculating the TTP; hence, the TTP may have been greater than 12 months at the 12-month follow-up. The rate of infertility was calculated using the following formula: Rate of infertility = (Number of couples with TTP ≥ 12 months + Number of couples who became pregnant using ART)/(Number of couples finally included in this study−Number of couples with TTP <12 months but not pregnant). Additionally, we collected information regarding conception type (natural pregnancy or ART).

### Covariates

Data regarding the following covariates were collected using a standardized questionnaire at baseline: both partners’ age (years), weight (kg), height (cm), education level (≤junior high school, high school or technical secondary school, ≥junior college), individual income (yuan/year), occupation (technical personnel, government official and clerk or enterprise clerk, businessperson, unemployed, manual worker, and others), smoking status (yes or no), drinking status (yes or no), physical activity levels (low, moderate, and high); age of menarche (<13 years or ≥13 years), pregnancy history (have or do not have), live birth history (have or do not have), spontaneous abortion history (have or do not have), stillbirth history (have or do not have), and induced abortion history (have or do not have); and age of first spermatogenesis (<15 years or ≥15 years). The BMI was calculated as weight/height^2^ (kg/m^2^). Smoking was defined as consuming at least one cigarette per day during the previous 6 months. Drinking was defined as consuming alcohol at least once during the previous 6 months. Participants’ physical activity level was evaluated based on the International Physical Activity Questionnaire Short Form Scale (Macfarlane *et al.*, 2007).

### Statistical analysis

Continuous data were expressed using mean (SD) or median (P_25_, P_75_), and categorical data were expressed using frequencies (proportions). Pregnant and non-pregnant groups were compared using the Student’s *t*-test or Kruskal–Wallis *H* test for the continuous variables and the chi-square test for the categorical variables.

First, we performed individual-specific analyses to assess the associations between each partner’s depression symptoms and the couple’s fertility. Second, considering the potential impact of shared depression in couples on their fertility, we performed couple-based analyses to assess the effects of co-exposure to depression in couples on fertility.

The probability of pregnancy in each month was estimated using the Kaplan–Meier method; the differences in the probability of pregnancy were evaluated using a log-rank test. Multivariable analyses with discrete time-to-event Cox regression were employed to estimate the fertility odds ratios (FORs) at different preconception depression levels, which are reported here along with their 95% CIs. Notably, FORs <1 indicate a longer time to become pregnant. Logistic regression was employed to estimate the association between depressive levels and infertility (dichotomous variable), with the results reported as risk ratios (RRs) along with their 95% CIs. The above two regression models were conducted in both individual-specific and couple-based analyses. In the individual-specific analysis, for women, data were adjusted for woman’s age, BMI, personal income, education level, occupation, smoking, drinking, physical activity levels, age of menarche, pregnancy history, live birth history, spontaneous abortion history, stillbirth history, and induced abortion history; for men, data were adjusted for man’s age, BMI, personal income, education level, occupation, smoking, drinking, physical activity levels, age of first spermatogenesis, age of first sexual intercourse; and their partner’s pregnancy history, live birth history, spontaneous abortion history, stillbirth history, and induced abortion history. In the couple-based analysis, data were adjusted for each partner’s age, BMI, personal income, education level, occupation, smoking, drinking, physical activity level; woman’s age of menarche, pregnancy history, live birth history, spontaneous abortion history, stillbirth history, and induced abortion history; and man’s age of first spermatogenesis and first sexual intercourse. Notably, we included couples who conceived through ART in the TTP analysis but classified them as the infertility group in the infertility analysis.

Considering that fertility starts dropping in women and men over 30 and 40 years of age, respectively (Schwartz and Mayaux, 1982; de La Rochebrochard *et al.*, 2006), we performed stratified analyses to assess whether the effect of depression on fertility was modified by maternal age (≤30 years or >30 years) and paternal age (<40 years or ≥40 years). Additionally, as maternal and paternal BMI exhibits a strong association with a couple’s fertility (Hernáez *et al.*, 2021), we examined whether the effect of depression on fertility was modified by the BMI for men and women (<18.5, <24.0, and ≥24.0 kg/m^2^). Moreover, we investigated the modification effects of a woman’s pregnancy history (have or do not have). Finally, we conducted three sensitivity analyses to assess our results’ robustness. First, we reanalyzed the relationship between depression and fertility by excluding the couples who became pregnant through ART. Second, we reanalyzed the relationship between depression and fertility in couples after excluding participants who were infertile owing to specific factors of infertility related to females or males or both, such as azoospermia, asthenospermia, blocked fallopian tubes, and PCOS. Third, the participants were recruited and followed up for over 3 years (2019–2022)—during the coronavirus disease 2019 (COVID-19) pandemic. This major public health event might have increased the risk of depression and infertility (COVID-19 Mental Disorders Collaborators, 2021; Somigliana *et al.*, 2021), hence, we conducted a sensitivity analysis among couples who believed that the COVID-19 pandemic did not affect their pregnancy preparedness.

The SPSS (version 23) software (IBM Crop, Armonk, NY, USA) was used to perform the statistical analyses. Finally, *α*-values <0.05 were considered statistically significant in all statistical tests.

## Results

### Descriptive analysis

Table 1 presents the demographic characteristics of included couples. The average age of women and men were 26.13 ± 3.50 and 27.13 ± 3.45 years, respectively. The women in the non-pregnant group were more likely to be older, be smokers, have a higher BMI, and have lower incomes, and less likely to have a history of spontaneous abortion, induced abortion, or pregnancy. The men in the non-pregnant group were more likely to be older and have an earlier age of first spermatogenesis. As Table 2 indicates, 11 668 (70.6%) couples achieved a pregnancy during the study period. Notably, 10 834 (65.6%) and 746 (4.5%) couples achieved pregnancy within the first 6 months and between the 6^th^ and 12^th^ months, respectively. The median (P_25_, P_75_) TTP was 3.0 (2.0, 6.0) months. The infertility rate was 13.01%.

**Table 1.** Baseline characteristics by pregnancy status based on the follow-up of 12 months (n = 16 521).

Baseline characteristics	Total (N = 16 521)	Pregnant (N = 11 668)	Non-pregnant (N = 4 853)	z/t/χ^2^	*P*
**Women**					
**Age (years)**	26.13 ± 3.50	25.93 ± 3.35	26.61 ± 3.80	**11.40**	**<0.001**
Missing (n)	8	4	4		
**BMI (kg/m^2^)**	22.20 ± 4.45	22.04 ± 4.41	22.58 ± 4.53	**6.97**	**<0.001**
Missing (n)	254	193	61		
**Education level**				0.820	0.664
≤ Junior high school	3920 (23.7)	2790 (23.9)	1130 (23.3)		
High school or technical secondary school	2560 (15.5)	1798 (15.4)	762 (15.7)		
≥ Junior college	10 041 (60.8)	7080 (60.7)	2961 (61.0)		
**Individual income (yuan/year)**				**14.80**	**0.001**
<30 000	4571 (27.7)	3234 (27.7)	1337 (27.5)		
≥30 000	6901 (41.8)	4965 (42.6)	1936 (39.9)		
≥60 000	5049 (30.6)	3469 (29.7)	1580 (32.6)		
**Occupation**				5.57	0.233
Technical personnel	2893 (17.8)	2043 (17.9)	850 (17.8)		
Government official and clerk or enterprise clerk	2640 (16.3)	1843 (16.1)	797 (16.7)		
Businessman	5147 (31.8)	3617 (31.6)	1530 (32.1)		
Unemployed	2480 (15.3)	1797 (15.7)	683 (14.3)		
Manual worker and other	3051 (18.8)	2138 (18.7)	913 (19.1)		
Missing (n)	310	230	80		
**Smoking**				**12.28**	**<0.001**
No	16 114 (97.5)	11 413 (97.8)	4701 (96.9)		
Yes	406 (2.5)	255 (2.2)	151 (3.1)		
Missing (n)	1	0	1		
**Drinking**				3.535	0.060
No	11 682 (72.0)	8292 (72.5)	3390 (71.0)		
Yes	4535 (28.0)	3151 (27.5)	1384 (29.0)		
Missing (n)	304	225	79		
**Age of menarche (years)**				1.163	0.281
<13	5821 (35.9)	4077 (35.6)	1744 (36.5)		
≥13	10 389 (64.1)	7360 (64.4)	3029 (63.5)		
Missing (n)	311	231	80		
**Physical activity levels**				1.469	0.480
Low	9247 (56.2)	6565 (56.5)	2682 (55.5)		
Moderate	5938 (36.1)	4166 (35.9)	1772 (36.7)		
High	1267 (7.7)	887 (7.6)	380 (7.9)		
Missing (n)	69	50	19		
**History of pregnancy**				**8.435**	**0.004**
Do not have	12 699 (76.9)	8897 (76.3)	3802 (78.3)		
Have	3822 (23.1)	2771 (23.7)	1051 (21.7)		
**History of live birth**				0.814	0.367
Do not have	15 598 (94.4)	11 004 (94.3)	4594 (94.7)		
Have	923 (5.6)	664 (5.7)	259 (5.3)		
**History of stillbirth**				0.391	0.532
Do not have	16 366 (99.1)	11 555 (99.0)	4811 (99.1)		
Have	155 (0.9)	113 (1.0)	42 (0.9)		
**History of spontaneous abortion**				**23.47**	**<0.001**
Do not have	15 819 (95.8)	11 115 (95.3)	4704 (96.9)		
Have	702 (4.2)	553 (4.7)	149 (3.1)		
**History of induced abortion**				**6.587**	**0.010**
Do not have	14 290 (86.5)	10 041 (86.1)	4249 (87.6)		
Have	2231 (13.5)	1627 (13.9)	604 (12.4)		
**Men**					
**Age (years)**	27.13 ± 3.45	26.96 ± 3.35	27.52 ± 3.64	**9.46**	**<0.001**
Missing (n)	16	12	4		
**BMI (kg/m^2^)**	23.85 ± 3.65	23.83 ± 3.63	23.92 ± 3.69	1.01	0.083
Missing (n)	257	188	69		
**Education level**				2.08	0.354
≤ Junior high school	3897 (23.6)	2758 (23.6)	1139 (23.5)		
High school or technical secondary school	3400 (20.6)	2432 (20.8)	968 (19.9)		
≥ Junior college	9224 (55.8)	6478 (55.5)	2746 (56.6)		
**Individual income (yuan/year)**				1.67	0.434
<60 000	5085 (30.8)	3589 (30.8)	1496 (30.8)		
≥60 000	6833 (41.4)	4858 (41.6)	1975 (40.7)		
≥100 000	4603 (27.9)	3221 (27.6)	1382 (28.5)		
**Occupation**				1.744	0.783
Technical personnel	5267 (32.5)	3720 (32.5)	1547 (32.4)		
Government official and clerk or enterprise clerk	1756 (10.8)	1237 (10.8)	519 (10.9)		
Businessman	4244 (26.2)	2993 (26.2)	1251 (26.2)		
Unemployed	558 (3.4)	407 (3.6)	151 (3.2)		
Manual worker and other	4377 (27.0)	3075 (26.9)	1302 (27.3)		
Missing (n)	319	236	83		
**Smoking**				0.000	0.990
No	8438 (51.1)	5959 (51.1)	2479 (51.1)		
Yes	8083 (48.9)	5709 (48.9)	2374 (48.9)		
**Drinking**				3.483	0.062
No	5699 (34.5)	3973 (34.1)	1726 (35.6)		
Yes	10 822 (65.5)	7695 (65.9)	3127 (64.4)		
**Age of first spermatogenesis (years)**				**3.888**	**0.049**
<15	8046 (49.7)	5620 (49.2)	2426 (50.9)		
≥15	8156 (50.3)	5812 (50.8)	2344 (49.1)		
Missing (n)	319	236	83		
**Physical activity levels**				3.944	0.139
Low	7086 (43.0)	4955 (42.6)	2131 (44.0)		
Moderate	6601 (40.1)	4717 (40.6)	1884 (38.9)		
High	2784 (16.9)	1960 (16.9)	824 (17.0)		
Missing (n)	50	36	14		

BMI, body mass index. Numbers in bold in the table indicates statistical significance, with a *P*-value <0.05. Pregnant and non-pregnant groups were compared using the Student’s *t*-test or Kruskal–Wallis H test for the continuous variables and the chi-square test for the categorical variables.

**Table 2.** The distribution of the cumulative pregnancy rate and median time to pregnancy in different groups.

N (%)		Couple				Men		Women	
**TTP** **(Months)**	**Total** **(N = 16 521)**	Neither partner with depression (N = 10 230)	Man-only depression (N = 2130)	Woman-only depression (N = 3088)	Both partners with depression (N = 1073)	No-depression (N = 13 318)	Depression (N = 3203)	No-depression (N = 12 360)	Depression (N = 4161)
≤1	3885 (23.5)	2434 (23.8)	489 (23.0)	717 (23.2)	245 (22.8)	3151 (23.7)	734 (22.9)	2923 (23.6)	962 (23.1)
≤2	6118 (37.0)	3825 (37.4)	772 (36.2)	1126 (36.5)	395 (36.8)	4951 (37.2)	1167 (36.4)	4597 (37.2)	1521 (36.6)
≤3	8332 (50.4)	5188 (50.7)	1070 (50.2)	1550 (50.2)	524 (48.8)	6738 (50.6)	1594 (49.8)	6258 (50.6)	2074 (49.8)
≤4	9095 (55.1)	5676 (55.5)	1157 (54.3)	1702 (55.1)	560 (52.2)	7378 (55.4)	1717 (53.6)	6833 (55.3)	2262 (54.4)
≤5	9621 (58.2)	6021 (58.9)	1218 (57.2)	1783 (57.7)	599 (55.8)	7804 (58.6)	1817 (56.7)	7239 (58.6)	2382 (57.2)
≤6	10 834 (65.6)	6790 (66.4)	1362 (63.9)	2009 (65.1)	673 (62.7)	8799 (66.1)	2035 (63.5)	8152 (66.0)	2682 (64.5)
<12	11 177 (67.7)	6988 (68.3)	1411 (66.2)	2074 (67.2)	704 (65.6)	9062 (68.0)	2115 (66.0)	8399 (68.0)	2778 (66.8)
12	11 580 (70.1)	7206 (70.4)	1477 (69.3)	2157 (69.9)	740 (69.0)	9363 (70.3)	2217 (69.2)	8683 (70.3)	2897 (69.6)
≤13	11 606 (70.2)	7219 (70.6)	1480 (69.5)	2163 (70.0)	744 (69.3)	9382 (70.4)	2224 (69.4)	8699 (70.4)	2907 (69.9)
≤15	11 639 (70.4)	7242 (70.8)	1482 (69.6)	2169 (70.2)	746 (69.5)	9411 (70.6)	2228 (69.6)	8724 (70.6)	2915 (70.1)
≤18	11 668 (70.6)	7257 (70.9)	1485 (69.7)	2178 (70.5)	748 (69.7)	9435 (70.8)	2233 (69.7)	8742 (70.7)	2926 (70.3)
MPT	3.0 (2.0,6.0)	3.0 (2.0,6.0)	3.0 (2.0,7.0)	3.0 (2.0,7.0)	3.0 (2.0,9.0)	3.0 (2.0,6.0)	3.0 (2.0,8.0)	3.0 (2.0,6.0)	3.0 (2.0,8.0)
CART	366 (2.22)	213 (58.2)	47 (12.8)	73 (19.9)	33 (9.0)	286 (78.1)	80 (21.9)	260 (71.0)	106 (29.0)
<12CART	315 (1.91)	186 (59.0)	43 (13.7)	59 (18.7)	27 (8.6)	245 (77.8)	70 (22.2)	229 (72.7)	86 (27.3)
≥12CART	51 (0.31)	27 (52.9)	4 (7.8)	14 (27.5)	6 (11.8)	41 (80.4)	10 (19.6)	31 (60.8)	20 (39.2)
<12 N-P	4034 (24.12)	2496 (61.9)	542 (13.4)	740 (18.3)	256 (6.3)	3236 (80.2)	798 (19.8)	3038 (75.3)	996 (24.7)

Pregnancy rate presented as N (%). TTP, time to pregnancy; MPT, median time to pregnancy (months); CART, conceived by ART; <12 CART, conceived by ART and with a TTP < 12 months; ≥12 CART, conceived by ART and with a TTP ≥ 12 months; <12 N-P, TTP <12 months and non-pregnant.

### Individual-specific analysis of associations between preconception depression and fertility

As Table 2 and Supplementary Fig. S1 indicate, for men, the median (P_25_, P_75_) TTP in the no-depression and depression groups was 3.0 (2.0, 6.0) and 3.0 (2.0, 8.0) months, respectively. The overall pregnancy rate in the no-depression group was significantly higher than that in the depression group (log-rank test: *P *=* *0.033). For women, the median (P_25_, P_75_) TTP in the no-depression and depression groups was 3.0 (2.0, 6.0) and 3.0 (2.0, 8.0) months, respectively. Additionally, the overall pregnancy rate in the no-depression group was significantly higher than that in the depression group (log-rank test: *P *=* *0.008).

**Figure 2.**
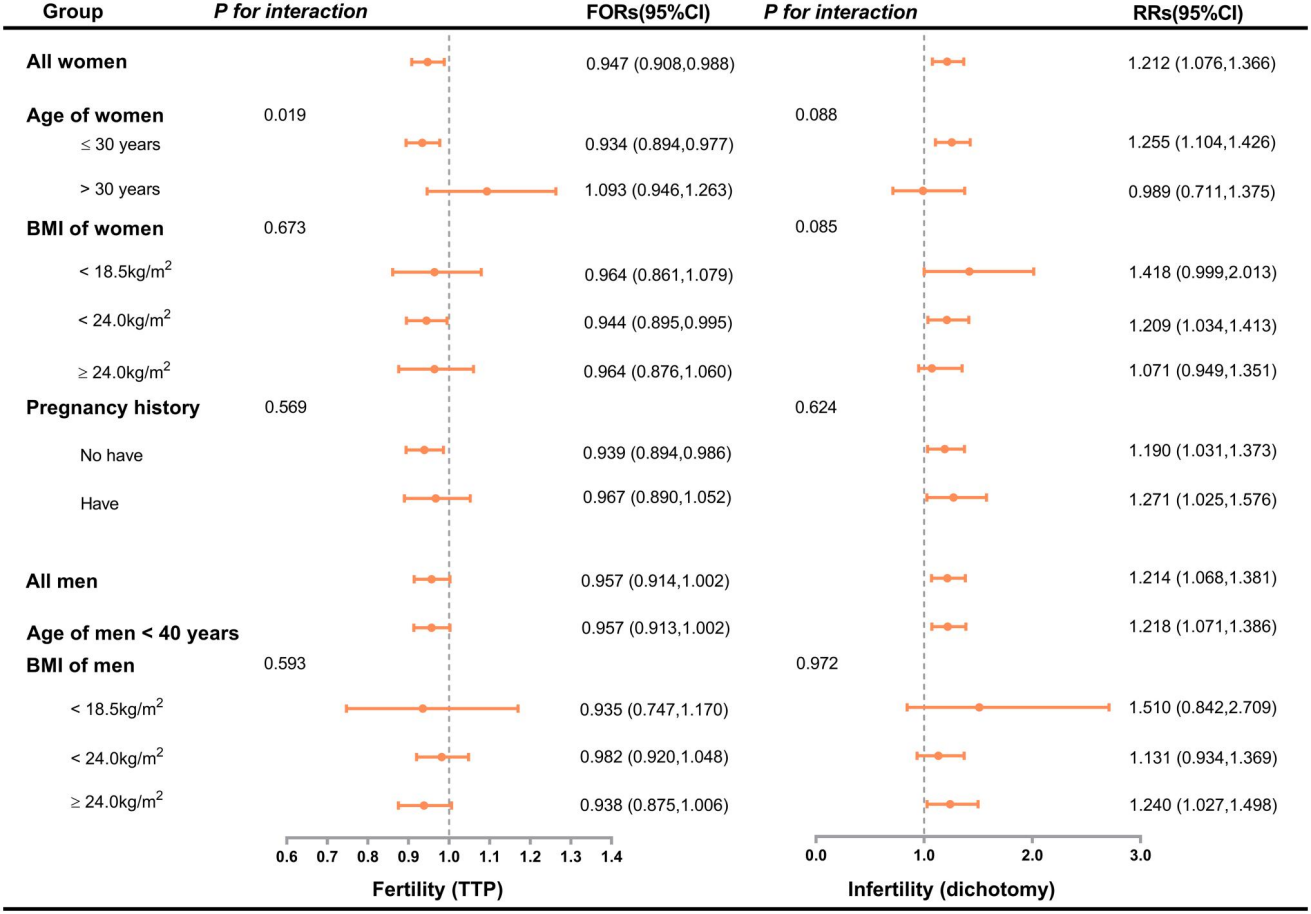
**Associations between each partner’s depression and couple fertility.** The no-depression group—defined as those with PHQ-9 scores <5—is the reference group. The depression group was defined as those with PHQ-9 scores ≥5. For women, data were adjusted for their age, BMI, personal income, education level, occupation, smoking, drinking, physical activity levels, age of menarche, pregnancy history, live birth history, spontaneous abortion history, stillbirth history, and induced abortion history. For men, data were adjusted for their age, BMI, personal income, education level, occupation, smoking, drinking, physical activity levels, age of first spermatogenesis, age of first sexual intercourse; their partner’s pregnancy history, live birth history, spontaneous abortion history, stillbirth history, and induced abortion history. In the subgroup analyses, data were adjusted for all the other factors, except the stratified factor. FOR, fertility odds ratio; RR, relative risk; PHQ-9, Patient Health Questionnaire-9; BMI, body mass index.

As Fig. 2 indicates, after adjusting for the potential confounders listed in the Materials and methods section, preconception depression in women was significantly related to reduced odds of fertility (FOR = 0.947, 95% CI: 0.908–0.988); and preconception depression in both men and women was significantly associated with an increased risk of infertility (women: RR = 1.212 [95% CI: 1.076–1.366]; men: RR = 1.214 [95% CI: 1.068–1.381]).

We further estimated the effect of individual preconception depression on couple fertility stratified by the woman’s age (≤30 years or >30 years), BMI (<18.5, <24.0, and ≥24.0 kg/m^2^), and pregnancy history (have or do not have); and the man’s age (<40 years or ≥40 years) and BMI (<18.5, <24.0, and ≥24.0 kg/m^2^). The woman’s age modified the associations between depression and couple fertility (*P* for interaction = 0.019); specifically, depression was significantly associated with reduced fertility in women ≤30 years (FOR = 0.934, 95% CI: 0.894–0.977), but no significant correlation was found between depression and fertility in women >30 years (FOR = 1.093, 95% CI: 0.946–1.263). Additionally, although the strength of the relationship between depression and fertility changed after the stratification analysis, we found no significant interaction between depression and other stratified factors (all *P* for interaction >0.05).

### Couple-based analysis of associations between preconception depression and fertility

As Table 2 indicates, the median (P_25_, P_75_) TTP in the four groups—namely, neither partner with depression, man-only depression, woman-only depression, and both partners with depression—were 3.0 (2.0, 6.0), 3.0 (2.0, 7.0), 3.0 (2.0, 7.0), and 3.0 (2.0, 9.0) months, respectively. The log-rank test revealed a significant difference in overall pregnancy rates between the above four groups (*P *=* *0.013). Specifically, couples where neither partner had depressive symptoms were most likely—while those wherein both the man and the woman had depressive symptoms were least likely—to conceive (Supplementary Fig. S1).

**Figure 3.**
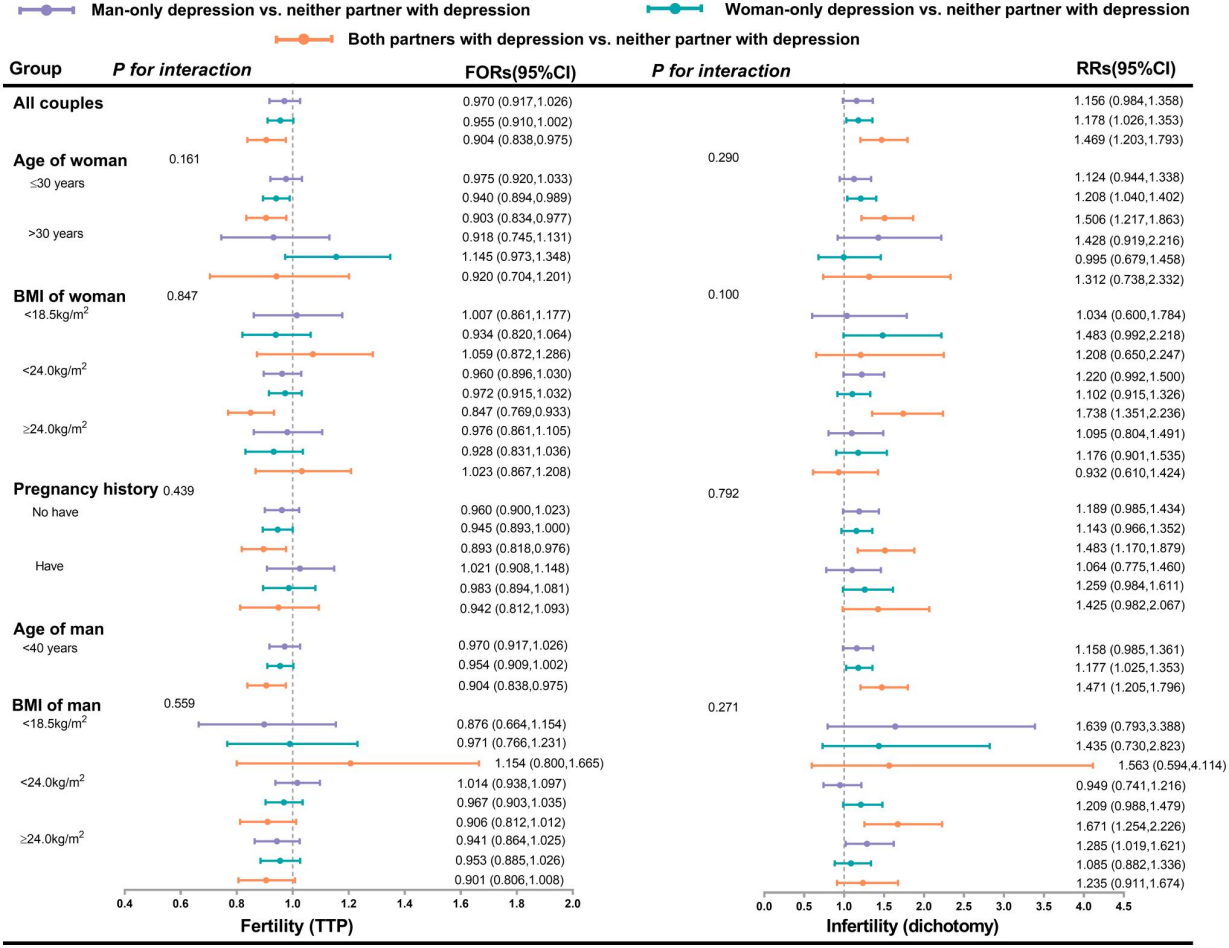
**Associations of couples’ depression with their fertility.** Data were adjusted for each partner’s age, BMI, personal income, education level, occupation, smoking, drinking, physical activity levels; women’s age of menarche, pregnancy history, live birth history, spontaneous abortion history, stillbirth history, and induced abortion history; and men’s age of first spermatogenesis and first sexual intercourse. In the subgroup analyses, data were adjusted for all the other factors, except the current stratified factor. FOR, fertility odds ratio; RR, relative risk; BMI, body mass index.

Figure 3 presents the full adjusted FOR (95% CI) and RR (95% CI) values for the association between couples’ depression and their fertility and infertility, respectively. For the fertility analysis, we adjusted for each partner’s age, BMI, personal income, education level, occupation, smoking, drinking, physical activity levels; the woman’s age of menarche, pregnancy history, live birth history, spontaneous abortion history, stillbirth history, and induced abortion history; and the man’s age of first spermatogenesis and first sexual intercourse (full adjusted model). We found that compared with couples where neither partner had depression, those where both partners had depression exhibited reduced fertility (adjusted FOR = 0.904, 95% CI: 0.838–0.975); meanwhile, no significant association was found in the man-only and woman-only depression groups. For the infertility analysis, in the fully adjusted model, compared to couples wherein neither partner had depression, the risk of infertility in the woman-only depression group and the group where both partners had depression increased by 17.8% (RR = 1.178; 95% CI: 1.026–1.353) and 46.9% (RR = 1.469; 95% CI: 1.203–1.793), respectively.

Figure 3 also presents the stratified analyses’ results. Stratifying factors exerted no significant modifying effect on the association between co-exposure to depression in couples and their fertility—with all *P* values for interaction greater than 0.05.

The above-mentioned results remained largely unchanged when we excluded the couples who became pregnant through ART and those with specific factors of infertility related to females or males or both or restricted the analysis to couples who believed that the COVID-19 pandemic did not affect their pregnancy preparedness (Supplementary Figs S2, S3, and S4).

## Discussion

Although much research has examined the relationship between depression and fertility, most of it has considered only one partner (Lynch *et al.*, 2012; Nillni *et al.*, 2016) or underrepresented populations (such as patients from reproductive centers) (Purewal *et al.*, 2018). In this prospective cohort study, we focused on the general population and considered both partners to explore the association between preconception depression and fertility. Our results indicated that compared to the couples wherein neither partner had depressive symptoms, the couples wherein both partners had depression needed a longer time to become pregnant and were more likely to be infertile. Our study—compared with previous studies—provided stronger evidence for a link between depression and fertility.

In this study, 10 834 (65.6%) and 11 580 (70.2%) couples achieved pregnancy within the first 6 and 12 months, respectively; this proportion is slightly lower than that reported by a community-based cohort study of TTP in Guangzhou, China, which indicated that 968 (69.1%) and 1082 (77.2%) couples conceived within the first 6 and 12 months, respectively (Zhong *et al.*, 2023). Our study reports an infertility rate of 13.01%, which falls within the range reported for the estimated period prevalence of infertility by the World Health Organization (10.0–16.4%) (https://www.who.int/health-topics/infertility). These similar pregnancy and infertility rates indicate that the participants are a good representation of the general population.

This study’s individual-specific analysis demonstrated that women with preconception depression needed a longer time to become pregnant, and women or men with preconception depression exhibited an increased risk of infertility. Consistent with our results, an internet-based preconception cohort with 2146 women found that participants with severe—compared with no or low—depressive symptoms at baseline exhibited a higher risk of decreased fertility (Nillni *et al.*, 2016). However, another prospective cohort with 339 women found no association between depression and TTP (in cycles) and the day-specific probabilities of pregnancy (Lynch *et al.*, 2012). Additionally, Evans-Hoeker *et al.* (2018) examined about 1600 couples who were infertile and pursuing non-IVF fertility treatments; they found that men with currently active major depression had low odds of achieving pregnancy but reported a positive association between women’s current active major depression and the odds of pregnancy. The inconsistency may be attributable to the differences in the study population, sample size, and study design.

Fertility depends on the couple, not merely on the man or the woman. Although couple-based epidemiological studies exploring the relationship between depressive symptoms and TTP and infertility are lacking, research has reported that depression decreases marital satisfaction and the frequency of sexual intercourse (Maroufizadeh *et al.*, 2018), which is closely associated with decreased fertility in couples. Moreover, we observed that even if the male partner had depressive symptoms and the female partner did not, their risk of infertility did not increase significantly. This indicates the different roles of women’s and men’s depression in infertility, highlighting that achieving a pregnancy involves more factors for women than of men. For example, depression is associated with multiple female subfecundity symptoms, including sexual dysfunction (McMillan *et al.*, 2017), menstrual disorder (Strine *et al.*, 2005), low rates of oocyte retrieval (Worly and Gur, 2015), and abnormal changes in reproductive hormones (Padda *et al.*, 2021). Additionally, female partners’ psychological states are strongly influenced by their male partners’ psychological states, but not vice versa. Studies have indicated that male partners’ depression is significantly associated with female partners’ happiness, but female partners’ depression is rarely associated with male partners’ happiness (Stimpson *et al.*, 2006). In sum, more couple-based investigations are needed to confirm our innovative findings.

Although we found that preconception depression was associated with decreased fertility and an increased risk of infertility, the underlying mechanisms remain unknown. Human reproduction depends on an intact HPG axis that includes multiple reproductive hormones, such as FSH, LH, and GnRH (Kim *et al.*, 2013; Maggi *et al.*, 2016). Notably, FSH and LH—controlled by the hypothalamus through GnRH—play an important role in promoting ovarian follicle development and maturation and prompting ovulation in women and initiating spermatogenesis in men (Marshall and Kelch, 1986). Previous studies have revealed a dysfunctional HPG axis in women with depression (Meller *et al.*, 2001), which may impair a couple’s fertility. Additionally, our research group has previously demonstrated that couples with—compared with those without—depression are more likely to engage in unhealthy lifestyles, such as smoking, drinking, and poor sleep quality, which are, reportedly, associated with decreased fertility (Sansone *et al.*, 2018; Hernáez *et al.*, 2022; Yao *et al.*, 2022). Moreover, patients with depression are more likely to consume antidepressants, which may lessen the odds of a woman with a history of depression becoming pregnant naturally (Casilla-Lennon *et al.*, 2016). Lastly, according to the actor–partner interdependence model, depression in one can decrease marital satisfaction in both partners (Maroufizadeh *et al.*, 2018), which may reduce their sexual intercourse frequency, thus decreasing their fertility (Zhu *et al.*, 2022).

Interestingly, our study revealed a significant moderating role of women’s age in the relationship between depressive symptoms in women and TTP, exhibiting a highly pronounced association in women aged less than 30 years but not in those aged above 30 years. Previous studies have indicated that women’s fertility begins declining significantly after the age of 30 years (Dunson *et al.*, 2002). Additionally, the decline in fertility is generally attributable to ovarian aging and other age-related gynecological diseases (Rowlands *et al.*, 2021; Moghadam *et al.*, 2022). Certainly, the sample size may have been reduced after stratification.

The strengths of this study include its large couple-based population (16 521 couples), multicenter design (16 premarital clinics), and representative sample of childbearing age (community-based sample), which were less influenced by hospital-based treatment processes and health-related biases, thereby allowing us to ascertain the associations with adequate power. Its further strengths include its consideration of both couple-based and individual-specific models, which enabled us to assess the effect of depression in men, women, and both partners on couple fertility.

Nevertheless, this study had several limitations. First, the self-reported nature of our data precipitates reporting and recall bias. Second, we employed a cut-off score of 5 to define depression—inconsistent with prior studies (Evans-Hoeker *et al.*, 2018), which used a cut-off score of 10 to define depression. However, the definition of depression with a cut-off score of 5 is ideal for community-based investigations. One study demonstrated a sensitivity of 0.86 and specificity of 0.75 for the cut-off score of 5 and a sensitivity of 0.49 and specificity of 0.95 for the cut-off score of 10 in primary care (Zuithoff *et al.*, 2010). Third, although we adjusted for as many confounding factors as possible, some residual confounding factors remain, such as the use of medications, especially depression medications, frequency of sexual intercourse, and prior depressive and anxiety symptoms. Fourth, we assessed depression only at baseline, but depression might change over time; therefore, we could not detect the effect of changes in depression over time on fertility.

## Conclusion

In this large couple-based prospective cohort study, individual-specific analysis revealed that preconception depression in either men or women was associated with an increased risk of infertility and that preconception depression in women was associated with a longer TTP, while couple-based analysis suggested that couples where both partners had depression needed a longer TTP and exhibit an increased risk of infertility. However, we found that even if the man had depressive symptoms while the woman did not, the couple’s risk of infertility did not significantly increase. Further evidence from well-designed epidemiological studies that consider medically diagnosed depression, assess depression at multiple timepoints, and employ longer follow-up periods are needed to clarify the effects of individual and couple preconception depression on their fertility.

## Supplementary Material

hoae032_Supplementary_Data

## Data Availability

The data underlying this article were provided by the Reproductive Health of Childbearing Couples-Anhui Cohort (RHCC-AC). Data will be shared on request to the corresponding author with permission of RHCC-AC.
