# Supplementary material for: Preconception depression reduces fertility: a couple-based prospective preconception cohort
Source: Hum Reprod Open. 2024 May 16;2024(3):hoae032. doi: 10.1093/hropen/hoae032 (PMC11150884; doi:10.1093/hropen/hoae032)
Supplement: hoae032_Supplementary_Data [file hoae032_supplementary_data.docx]

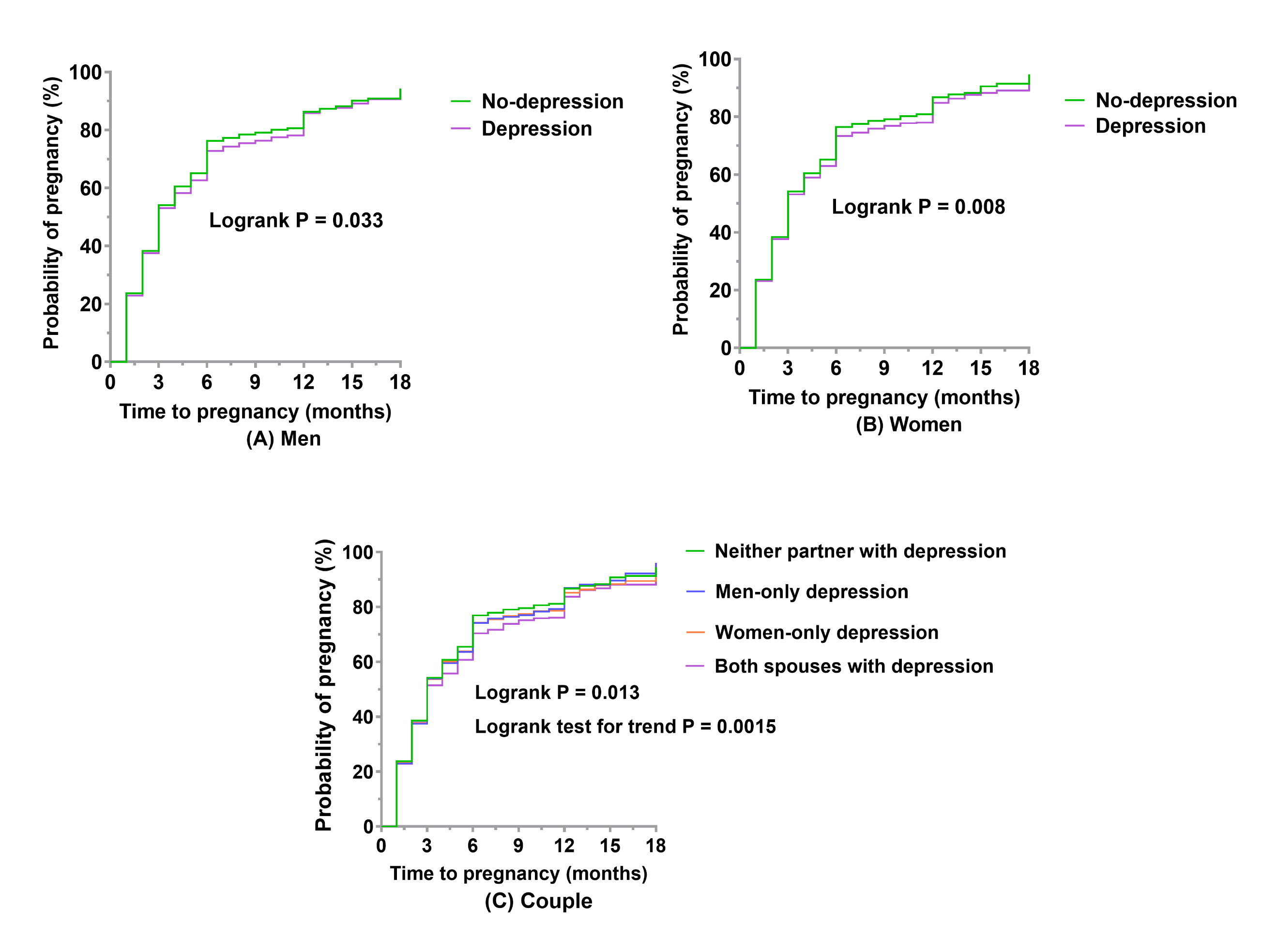


**Supplementary Figure S1** Kaplan–Meier survival curves for probability of pregnancy.


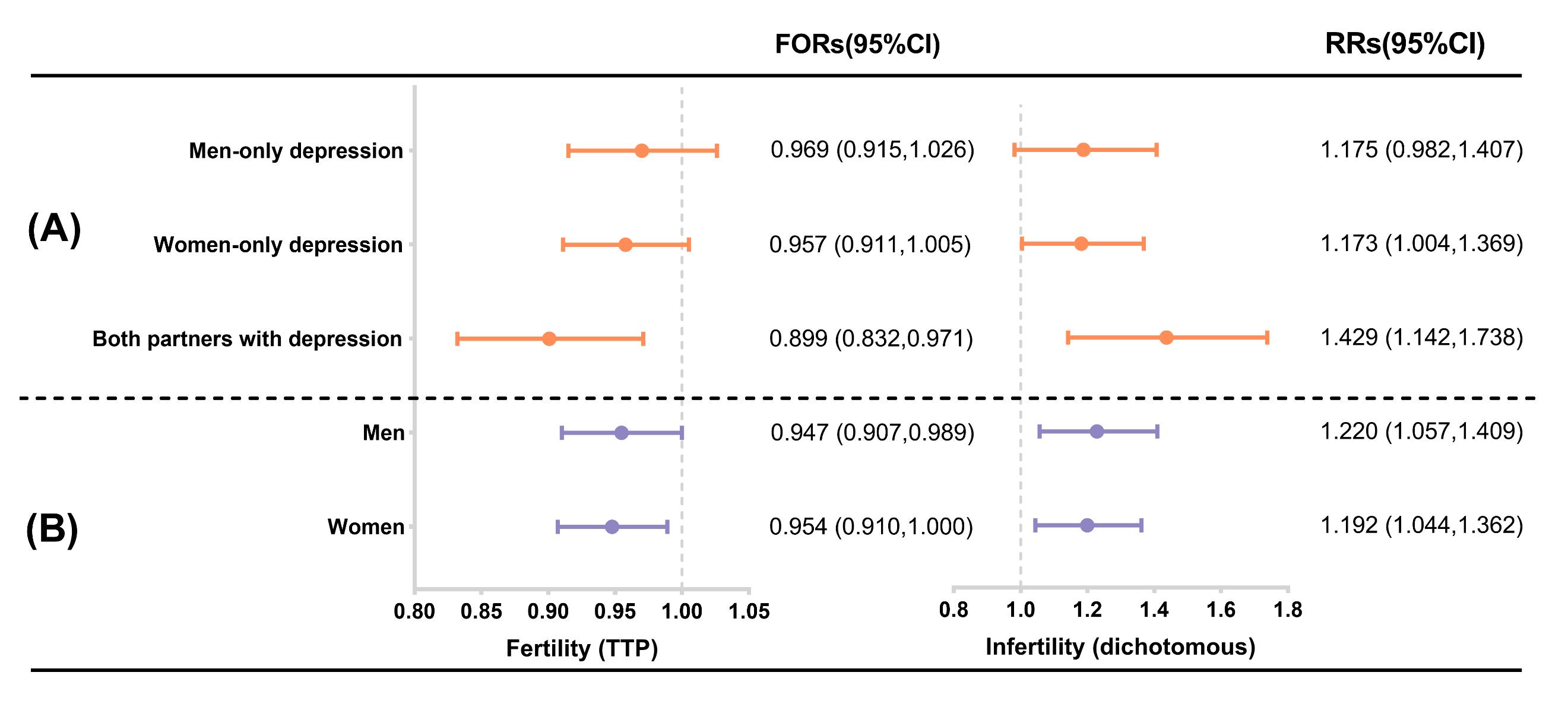


**Supplementary Figure S2** The associations between preconception depression and fertility when excluded the couples who became pregnant through assisted reproductive treatment.

**(A)** Couple-based analyses: The group of couples wherein neither partner had depression is the reference group. Data were adjusted for each partner’s age, BMI, personal income, educational level, occupation, smoking, drinking, physical activity levels; women’s age of menarche, pregnancy history, live birth history, spontaneous abortion history, stillbirth history, and induced abortion history; and men’s age of first spermatogenesis and first sexual intercourse. **(B)** Individual-specific analyses: the no-depression group—defined as those with PHQ-9 scores <5—is the referene group. The depression group was defined as those with PHQ-9 scores ≥5. For women, data were adjusted for their age, BMI, personal income, educational level, occupation, smoking, drinking, physical activity levels, age of menarche, pregnancy history, live birth history, spontaneous abortion history, stillbirth history, and induced abortion history. For men, data were adjusted for their age, BMI, personal income, educational level, occupation, smoking, drinking, physical activity levels, age of first spermatogenesis, age of first sexual intercourse; their partner’s pregnancy history, live birth history, spontaneous abortion history, stillbirth history, and induced abortion history. FOR: fertility odds ratio, RR: relative risk, BMI: body mass index.


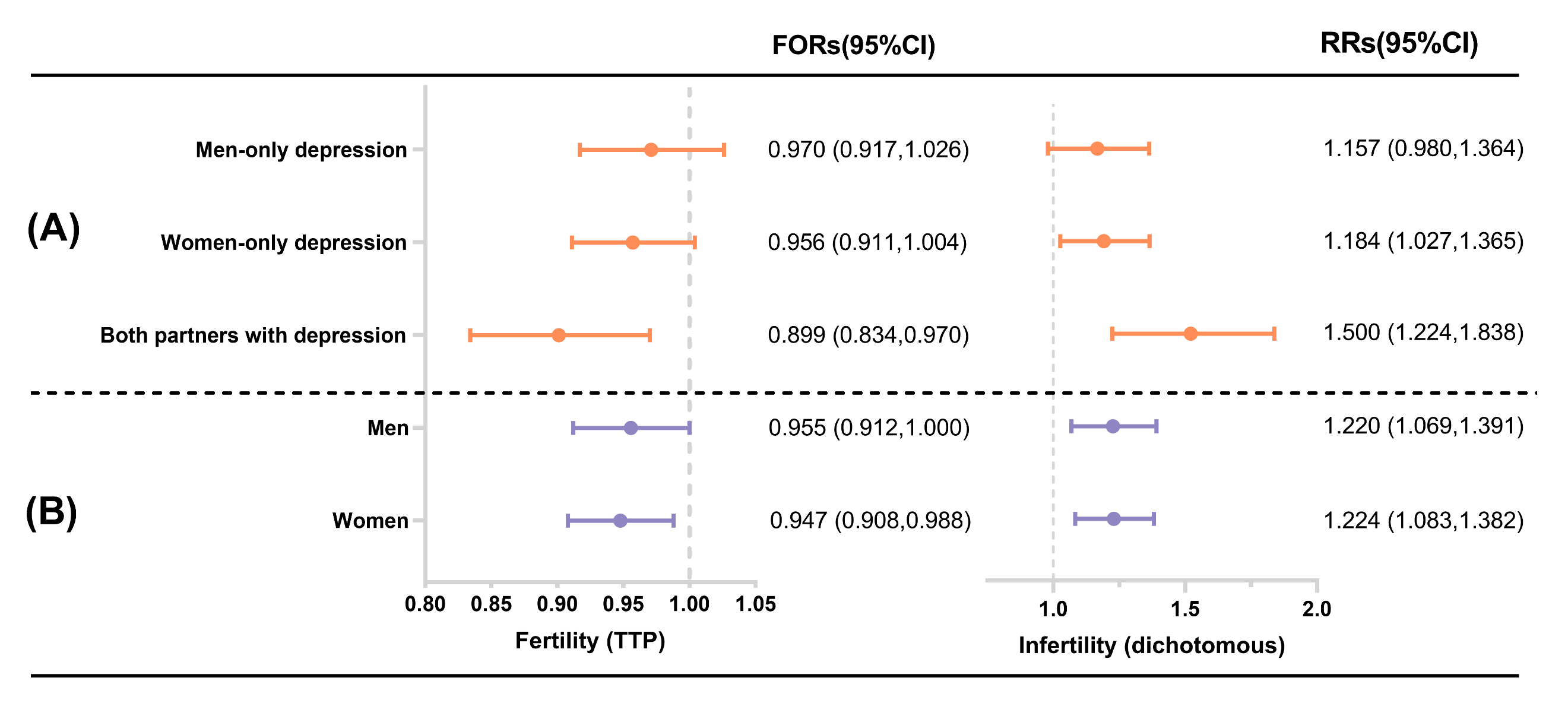


**Supplementary Figure S3** The associations between preconception depression and fertility when excluded the couples with specific factors of infertility related to females or males or both.

**(A)** Couple-based analyses: The group of couples wherein neither partner had depression is the reference group. Data were adjusted for each partner’s age, BMI, personal income, educational level, occupation, smoking, drinking, physical activity levels; women’s age of menarche, pregnancy history, live birth history, spontaneous abortion history, stillbirth history, and induced abortion history; and men’s age of first spermatogenesis and first sexual intercourse. **(B)** Individual-specific analyses: the no-depression group—defined as those with PHQ-9 scores <5—is the referene group. The depression group was defined as those with PHQ-9 scores ≥5. For women, data were adjusted for their age, BMI, personal income, educational level, occupation, smoking, drinking, physical activity levels, age of menarche, pregnancy history, live birth history, spontaneous abortion history, stillbirth history, and induced abortion history. For men, data were adjusted for their age, BMI, personal income, educational level, occupation, smoking, drinking, physical activity levels, age of first spermatogenesis, age of first sexual intercourse; their partner’s pregnancy history, live birth history, spontaneous abortion history, stillbirth history, and induced abortion history. FOR: fertility odds ratio, RR: relative risk, BMI: body mass index.


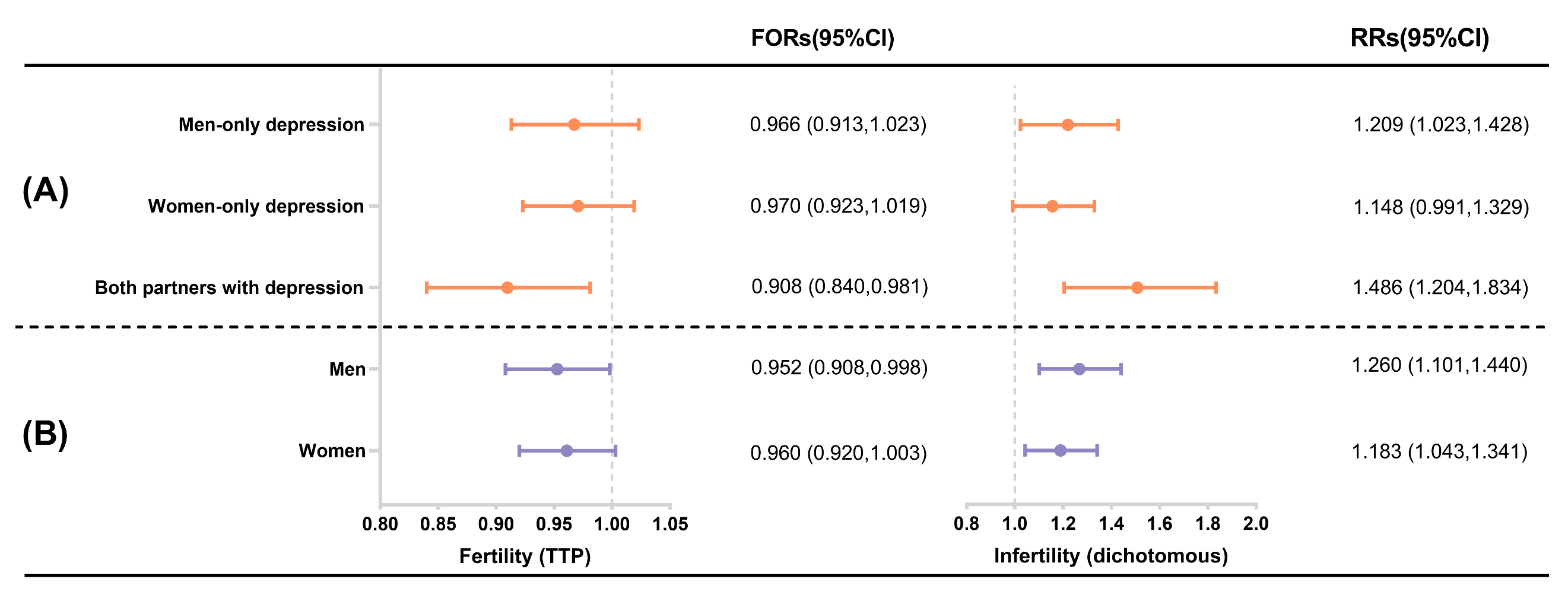


**Supplementary Figure S4** The associations between preconception depression and fertility when restricted the analysis to couples who believed that the COVID-19 pandemic did not affect their pregnancy preparedness.

**(A)** Couple-based analyses: The group of couples wherein neither partner had depression is the reference group. Data were adjusted for each partner’s age, BMI, personal income, educational level, occupation, smoking, drinking, physical activity levels; women’s age of menarche, pregnancy history, live birth history, spontaneous abortion history, stillbirth history, and induced abortion history; and men’s age of first spermatogenesis and first sexual intercourse. **(B)** Individual-specific analyses: the no-depression group—defined as those with PHQ-9 scores <5—is the referene group. The depression group was defined as those with PHQ-9 scores ≥5. For women, data were adjusted for their age, BMI, personal income, educational level, occupation, smoking, drinking, physical activity levels, age of menarche, pregnancy history, live birth history, spontaneous abortion history, stillbirth history, and induced abortion history. For men, data were adjusted for their age, BMI, personal income, educational level, occupation, smoking, drinking, physical activity levels, age of first spermatogenesis, age of first sexual intercourse; their partner’s pregnancy history, live birth history, spontaneous abortion history, stillbirth history, and induced abortion history. FOR: fertility odds ratio, RR: relative risk, BMI: body mass index.
